# Hemostatic function remains unaltered following therapeutic factor XIIa inhibition with garadacimab

**DOI:** 10.1016/j.rpth.2026.106993

**Published:** 2026-09-29

**Authors:** Danny M. Cohn, Henriette Farkas, Marc A. Riedl, Harsha Shetty, John-Philip Lawo, Maressa Pollen, H. Henry Li

**Affiliations:** 1 Department of Vascular Medicine, Amsterdam Cardiovascular Sciences, Amsterdam UMC, University of Amsterdam, Amsterdam, The Netherlands; 2 Department of Internal Medicine and Haematology, Hungarian Angioedema Center of Reference and Excellence, Semmelweis University, Budapest, Hungary; 3 University of California San Diego, San Diego, California, USA; 4 CSL Behring, King of Prussia, Pennsylvania, USA; 5 CSL Innovation GmbH, Marburg, Germany; 6 Institute for Asthma and Allergy, Chevy Chase, Maryland, USA

**Keywords:** angioedemas, hereditary, antibodies, monoclonal, bleeding, factor XIIa, monoclonal

## Abstract

**Background:**

Activated factor XII (FXIIa) initiates the kallikrein–kinin system, which is dysregulated in hereditary angioedema (HAE), leading to overproduction of bradykinin, the key mediator of swelling in HAE. Garadacimab (an anti-FXIIa monoclonal antibody) was recently approved for HAE long-term prophylaxis. In previous publications, patients with congenital factor XII (FXII) deficiency reported no increased risk of bleeding. The effect of long-term FXIIa inhibition on hemostasis in individuals with increased bleeding risk is not fully understood.

**Objectives:**

To assess the impact of FXIIa inhibition on hemostasis in patients with increased bleeding risk receiving garadacimab.

**Methods:**

Abnormal bleeding events were monitored and medically reviewed in patients with HAE (phase 2, pivotal phase 3, and phase 3 open-label extension [OLE] studies) who received garadacimab with or without concomitant anticoagulant/antiplatelet medications. Abnormal bleeding events were further evaluated in patients who underwent invasive surgical/medical procedures in the phase 3 OLE and by concurrent Standardized MedDRA Query analysis across HAE studies. Relevant laboratory markers were evaluated in the phase 3 OLE. Abnormal bleeding was also assessed in patients with severe COVID-19 (phase 2 study) who received garadacimab with or without concomitant anticoagulant/antiplatelet medications.

**Results:**

No garadacimab-related abnormal bleeding events were observed, including among patients who received concomitant anticoagulant/antiplatelet medications or underwent invasive surgical/medical procedures. Plasma coagulation assays reflected natural variation.

**Conclusion:**

Garadacimab-mediated FXIIa inhibition had no impact on hemostasis in patients with HAE or COVID-19 and increased bleeding risk, consistent with patients with congenital FXII deficiency who report no bleeding phenotypes.

## Essentials

• Garadacimab, an activated factor XII (FXIIa) inhibitor, is approved for hereditary angioedema.
• This post hoc analysis assessed the impact of FXIIa inhibition with garadacimab on hemostasis.
• No garadacimab-related abnormal bleeding was reported in patients with increased bleeding risk.
• Long-term garadacimab-mediated FXIIa inhibition is not associated with increased bleeding risk.

## 1 Introduction

Factor XII (FXII), or Hageman factor, is the principal initiator of the contact system, encompassing the kallikrein–kinin system (KKS), fibrinolytic pathway, intrinsic coagulation system, and complement cascade [1], [2], [3]. The KKS, regulated by C1 inhibitor (C1INH), is initiated upon FXII autoactivation to activated factor XII (FXIIa) to cleave prekallikrein into plasma kallikrein, resulting in bradykinin production [1], [2], [3].

Hereditary angioedema (HAE) is a rare genetic disease primarily caused by C1INH deficiency or dysfunction, resulting in KKS dysregulation and increased bradykinin production, which leads to recurrent, unpredictable, debilitating, and potentially life-threatening attacks of angioedema throughout the lifetime of affected individuals [2], [3], [4].

The roles of FXII in the intrinsic coagulation pathway have been explored in animals and humans; patients with congenital FXII deficiency exhibited no increased risk of bleeding [5], [6], [7], [8].

Garadacimab is an anti-FXIIa monoclonal antibody that potently inhibits activated FXII at a dose of 10 mg/kg [9]. Garadacimab 200 mg once monthly is indicated for HAE long-term prophylaxis in patients aged ≥12 years based on the early, durable, and sustained protection against HAE attacks and favorable long-term safety profile reported throughout the HAE clinical program, including phase 2 (NCT03712228), pivotal phase 3 (VANGUARD; NCT04656418), and ongoing phase 3 open-label extension (OLE; NCT04739059) studies [10], [11], [12], [13], [14], [15]. No increase in bleeding events is typically reported in patients with HAE [16]. However, due to the role of FXIIa in the intrinsic coagulation and fibrinolytic pathways, abnormal bleeding events and thromboembolic events were monitored as adverse events of special interest (AESIs) per protocol throughout the garadacimab clinical program [10], [11], [12], [16]. Clinically significant abnormalities in laboratory parameters of coagulation were also assessed [10], [11], [12].

The most common treatment-emergent adverse events (AEs) across HAE studies of garadacimab were injection-site reactions [10], [11], [12]. Importantly, no garadacimab-related abnormal bleeding events were reported [10], [11], [12].

Another phase 2 study investigated the efficacy and safety of a single garadacimab dose to prevent disease worsening in patients hospitalized with severe COVID-19 alongside standard-of-care treatment [17]. The primary efficacy endpoint was not met in this study. Hemostasis-related safety is of interest because COVID-19 pathology may contribute to disturbances of the coagulation system, and the use of heparins to manage COVID-19 may further increase bleeding risk [18], [19].

Although no safety signals were identified in these studies, physicians may encounter patients at high risk of bleeding in clinical practice. To better inform these clinicians, in-depth subgroup analyses were performed in patients at increased risk of bleeding, including those with HAE or severe COVID-19, those receiving concomitant medications associated with increased bleeding risk, and those who underwent surgical/medical procedures.

## 2 Methods

### 2.1 Data analyses

According to the schedule of assessments for each clinical study and in accordance with Good Clinical Practice standards, untoward events that occurred after informed consent were recorded as AEs, including any significant laboratory abnormalities throughout the study duration. Investigator-reported abnormal bleeding events were monitored based on clinical judgment as AESIs per protocol in the HAE phase 2, pivotal phase 3, and phase 3 OLE studies (data cutoff: June 2023) and were therefore evaluated, medically reviewed, and assessed to establish severity, resolution, cause, and treatment-relatedness. Of note, patients with a prior history of clinically significant arterial or venous thrombosis were excluded from the HAE phase 2 study but not from the HAE phase 3 or phase 3 OLE studies. Additionally, in the phase 3 OLE, abnormal bleeding events were evaluated post hoc per study protocol in patients who received garadacimab and also underwent concomitant invasive surgical/medical procedures associated with increased bleeding risk. AEs were also medically reviewed in all HAE studies of patients receiving garadacimab and concomitant anticoagulant/antiplatelet medications.

A prespecified Standardized MedDRA Query (SMQ) search was performed as part of the routine signal detection process using the query “hemorrhages” in parallel to the HAE studies to identify potential abnormal bleeding events that were not reported by study investigators and assess their clinical relevance or causal association with garadacimab. SMQ-identified hits were medically evaluated for clinical context and relatedness, including company and investigator assessments.

Changes in relevant laboratory markers of coagulation, including activated partial thromboplastin time (aPTT) and prothrombin time, were monitored irrespective of the incidence of AEs and evaluated at months 12, 18, and 24 vs run-in in the HAE phase 3 OLE.

In a separate analysis of patients without HAE with severe COVID-19, abnormal bleeding events were assessed in those who received garadacimab with or without concomitant anticoagulant/antiplatelet medications.

The designs and detailed demographic characteristics of patients in the HAE and COVID-19 studies have been described previously (study designs are summarized in Table 1) [10], [11], [12], [17].

**Table 1:** Study designs and enrollment.

Study	HAE				COVID-19
	Phase 2 (TP1) (garadacimab *n* = 24, placebo *n* = 8)	Phase 2 OLE (TP2) (garadacimab *n* = 24, placebo *n* = 8)	Pivotal phase 3 (garadacimab *n* = 39, placebo *n* = 25)	Phase 3 OLE^a^ (garadacimab *n* = 161)	Phase 2 (garadacimab + SOC *n* = 58, placebo + SOC *n* = 59)
Study type	Multicenter, randomized, double-blind, placebo-controlled	Multicenter, open-label	Multicenter, randomized, double-blind, placebo-controlled	Multicenter, open-label, single-arm	Multicenter, randomized, double-blind, placebo-controlled, parallel-group
Inclusion criteria					
Patients	Patients with HAE-C1INH or HAE-nC1INH	Patients with HAE-C1INH or HAE-nC1INH	Patients with HAE-C1INH	Patients with HAE-C1INH or HAE-nC1INH^b^	Patients hospitalized with severe COVID-19^c^
Age, years	≥18	≥18	≥12	≥12	≥18
Study drug(s)					
Loading dose(s)	Garadacimab IV loading dose 40 mg, 100 mg, or 300 mg; or placebo	No loading dose	Garadacimab 400 mg SC loading dose (2 × 200 mg injections)	Garadacimab 400 mg SC loading dose (2 × 200 mg injections) for newly enrolled patients	No loading dose
Study dose(s)	Garadacimab 75 mg SC once monthly, 200 mg SC once monthly, or 600 mg SC once monthly; or placebo, R 1:1:1:1	Garadacimab 200 mg SC once monthly^d^	Garadacimab 200 mg SC once monthly or placebo, R 3:2	Garadacimab 200 mg SC once monthly	Garadacimab 700 mg IV single dose or placebo, R 1:1^e^
Primary endpoint	Time-normalized monthly number of attacks for patients receiving garadacimab 200 mg or 600 mg	Time-normalized monthly number of attacks (supportive analysis to the primary endpoint in TP1)	Time-normalized monthly number of attacks	TEAEs	Incidence of progression to tracheal intubation or death
Reference	Craig et al. 2022 [12]	Craig et al. 2024 [20]	Craig et al. 2023 [10]	Reshef et al. 2024 [11]	Papi et al. 2023 [17]

HAE, hereditary angioedema; HAE-C1INH, hereditary angioedema due to C1 inhibitor deficiency or dysfunction; HAE-nC1INH, hereditary angioedema with normal C1 inhibitor; IV, intravenous; OLE, open-label extension; R, randomized; SC, subcutaneous; SOC, standard of care; TEAE, treatment-emergent adverse event; TP, treatment period.

a Included rollover patients who previously received garadacimab in the phase 2 or phase 3 studies, garadacimab-naïve patients who received placebo in the pivotal phase 3 study, and newly enrolled garadacimab-naïve patients.

b Patients who were enrolled in the phase 2 study with HAE-nC1INH were able to roll over into the phase 3 OLE, but patients with HAE-nC1INH were not eligible to be newly enrolled.

c Severe COVID-19 was defined as SARS-CoV-2 confirmed by polymerase chain reaction with interstitial pneumonia confirmed on chest computed tomography or X-ray.

d Patients who originally received garadacimab 75 mg once monthly, open-label garadacimab, or placebo were randomized to garadacimab 200 mg or 600 mg once monthly, and after protocol amendment, all patients who were receiving garadacimab 600 mg once monthly were downtitrated to 200 mg once monthly.

e 28-day follow-up period post-dose.

### 2.2 Statistical analysis

AE analyses were performed as part of the routine signal detection process throughout the respective study protocols in patients with HAE or COVID-19, including the exploration of AESIs per protocol and SMQ analysis; events in high-risk populations were evaluated post hoc. Bleeding events were summarized descriptively.

### 2.3 Ethics statement

All studies were performed in accordance with the International Council for Harmonisation of Technical Requirements for Pharmaceuticals for Human Use Good Clinical Practice guidelines, all applicable national and local regulations, and CSL Behring standard operating procedures, as published previously [10], [11], [12], [17].

## 3 Results and Discussion

### 3.1 No garadacimab-related abnormal bleeding events were reported across the HAE clinical program to date

Across the garadacimab clinical program to date, encompassing 172 patients receiving garadacimab, one patient experienced an isolated episode of mild epistaxis in the OLE period of the phase 2 study with 600 mg garadacimab (median exposure, 10.1 months); this was deemed unrelated to garadacimab by the investigator, had no identifiable cause, and resolved without medical intervention. The patient experienced no recurrence, no additional bleeding events, or any abnormalities in prothrombin time and continued treatment for >5 years, with almost 3 years at the approved 200 mg once-monthly dose and no further abnormal bleeding-related events were reported.

### 3.2 No abnormal bleeding events were reported in patients with HAE and high bleeding risk who received garadacimab

During the HAE phase 3 OLE (*n* = 161), 25 patients receiving garadacimab underwent a total of 35 concomitant invasive surgical/medical procedures and no abnormal bleeding events were reported. Dental procedures were the most common (*n* = 16, 45.7%), followed by ophthalmologic surgeries (*n* = 5, 14.3%), endoscopic investigations (*n* = 3, 8.6%), tumor removals (*n* = 2, 5.7%), muscle repair surgeries (*n* = 2, 5.7%), toe surgeries (*n* = 2, 5.7%), partial lung resection (*n* = 1, 2.9%), cutaneous cyst surgery (*n* = 1, 2.9%), nevus removal (*n* = 1, 2.9%), portacath removal (*n* = 1, 2.9%), and biopsy (*n* = 1, 2.9%). Overall, 7 (28.0%) patients used short-term prophylaxis (STP), and 3 (12.0%) used on-demand treatment. Two patients reported HAE attacks following tooth extractions, both of whom received STP.

AE data for patients receiving concomitant anticoagulant/antiplatelet medications were pooled from the HAE studies (these medications were not permitted in the phase 2 study). In the pivotal phase 3 study, 4 patients received concomitant aspirin (2 patients), ticagrelor, or apixaban. In the phase 3 OLE, a total of 20 patients received concomitant anticoagulant/antiplatelet medications: 13 received aspirin (isolated dose: 5; ongoing basis: 8), 2 received ongoing apixaban, 1 received ongoing ticagrelor, and 4 received isolated doses of enoxaparin (*n* = 2), mucopolysaccharide polysulfuric acid ester (*n* = 1), or heparinoid (*n* = 1). Investigators reported bleeding events in 1 patient in the phase 2 OLE treatment period (mild epistaxis unrelated to treatment), none in the pivotal phase 3 study, and 2 in the phase 3 OLE (vaginal hemorrhage and contusion [bruised right toe]; both occurred within 1 to 2 months of aspirin administration and both were deemed not garadacimab-related by the investigator).

### 3.3 No clinically relevant abnormal bleeding events were identified by SMQ analysis in the garadacimab HAE clinical program

To investigate the potential effect of long-term FXIIa inhibition more thoroughly, SMQ analysis was performed alongside the HAE clinical program. The term “hemorrhages” yielded 25 hits (Table 2). The exposure-adjusted event rate was 0.089 events per patient-year for garadacimab (*n* = 172) and <0.22 events per patient for placebo during the placebo-controlled treatment period (TP1) of the phase 2 (*n* = 8) and the pivotal phase 3 (*n* = 25) studies.

**Table 2:** Treatment exposure for patients included in the SMQ analysis.

Study	Phase 2 (garadacimab *n* = 24, placebo *n* = 8)	Pivotal phase 3 (garadacimab *n* = 39, placebo *n* = 25)	Phase 3 OLE (garadacimab *n* = 161)
Number of hits (n)	11 (7)	2 (2)	12 (10)
Median exposure, months	TP1: 2.98 TP2: 23.89^a^	5.98	13.80
Total exposure, patient-years	71.89	19.43	184.57

OLE, open-label extension; SMQ, Standardized MedDRA Query; TP, treatment period.

a Duration of TP1 was 12 weeks; duration of TP2 was ≥44 weeks and reflects the total treatment duration.

SMQ analysis identified 21 bleeding events: contusion (10, *n* = 8), traumatic hematoma (3, *n* = 2), hematuria (2, *n* = 1), epistaxis (2, *n* = 2), intermenstrual bleeding (1), occult blood in urine (1), vaginal hemorrhage (1), and blood extravasation (1). One epistaxis event was reported as an AESI per protocol by investigators (HAE phase 2 study: mild, resolved, unrelated to garadacimab). All events were deemed unrelated to garadacimab by investigators and sponsors upon medical review; most were mild, all patients recovered, and no clinically relevant abnormalities in coagulation parameters were reported.

Four injection-site reactions (3 bruising, 1 hematoma) were identified but not classified as bleeding events.

### 3.4 No abnormal bleeding events were reported in patients with severe COVID-19 at high risk of bleeding who received garadacimab

A substantial proportion of patients hospitalized with severe COVID-19 (94.8%, *n* = 55/58) received concomitant anticoagulant/antiplatelet medications at the time of garadacimab infusion or during follow-up (Table 3). Overall, 7 bleeding events were reported, 3 with garadacimab vs 4 with placebo. All 3 events reported with garadacimab were epistaxis, deemed treatment-unrelated by investigators: 2 cases were mild and resolved and 1 was severe and did not resolve during follow-up. Epistaxis, sometimes severe, is a known complication of COVID-19, and each case was considered most consistent with the underlying disease, in accordance with investigators' assessment that the events were unrelated to garadacimab [21].

**Table 3:** Bleeding events identified in patients who received concomitant anticoagulant/antiplatelet medication in the garadacimab phase 2 COVID-19 study.

Study	Concomitant medication	Duration of administration	Bleeding events
Phase 2 COVID-19 (*n* = 55)	Enoxaparin (*n* = 33) Aspirin + enoxaparin (*n* = 10) Aspirin + enoxaparin + heparin (*n* = 3) Aspirin (*n* = 2) Enoxaparin + apixaban (*n* = 2) Enoxaparin + heparin + apixaban (*n* = 1) Enoxaparin + heparin + apixaban + aspirin (*n* = 1) Enoxaparin + heparin + epoprostenol (*n* = 1) Enoxaparin + rivaroxaban (*n* = 1) Heparin (*n* = 1)	All administered at the time of garadacimab infusion or during follow-up	Three epistaxis events with garadacimab (deemed unrelated by investigators)^a^ Four events in 1 patient receiving placebo (hemorrhagic stroke, subarachnoid hemorrhage, thrombocytopenia, and subdural hematoma)

a One patient experienced a serious event of severe epistaxis 11 days after starting prophylactic enoxaparin, which remained unresolved prior to their death (due to COVID-19). One patient who had been treated with prophylactic aspirin for 5 years experienced mild epistaxis on one occasion, which resolved on the same day, and received a 4-day course of enoxaparin starting the day after epistaxis. One patient who had been treated with prophylactic aspirin for 3 years received prophylactic enoxaparin for 6 days, followed by 6 days without treatment in addition to aspirin, then heparin for 2 days in response to adverse events and a 1-week course of apixaban starting after that second day; 2 days into the course of apixaban, the patient experienced a mild epistaxis that resolved after 9 days. All treatments were dosed according to standard clinical practice. All events were deemed unrelated to garadacimab.

### 3.5 Trends in laboratory markers reflect natural variation, suggesting no impact on hemostasis

In a murine FeCl_3_-induced thrombosis model, the garadacimab parenteral antibody (3F7) did not affect bleeding risk or prothrombin time; however, dose-dependent aPTT prolongation was observed, an expected on-target effect of FXIIa inhibition with the aPTT assay [13], [14], [22]. FXIIa inhibition-associated aPTT prolongation was observed in patients who received garadacimab 600 mg in the phase 2 study, but was not accompanied by elevation in other coagulation parameters or associated with clinical signs of abnormal bleeding or thrombosis [23]. Furthermore, no clinical signs of abnormal bleeding or impact on prothrombin time were observed in phase 1 and phase 2 studies with garadacimab 600 mg subcutaneously or 700 mg intravenously, or in Japanese and White participants in a pharmacokinetic analysis [9], [10], [12], [23].

In this analysis of the phase 3 OLE, all relevant laboratory markers remained unchanged vs run-in for patients receiving garadacimab 200 mg (Figure 1A, B). Trends in these laboratory markers were as expected for patients with HAE pathophysiology; no abnormality was evident.

**Figure 1:**
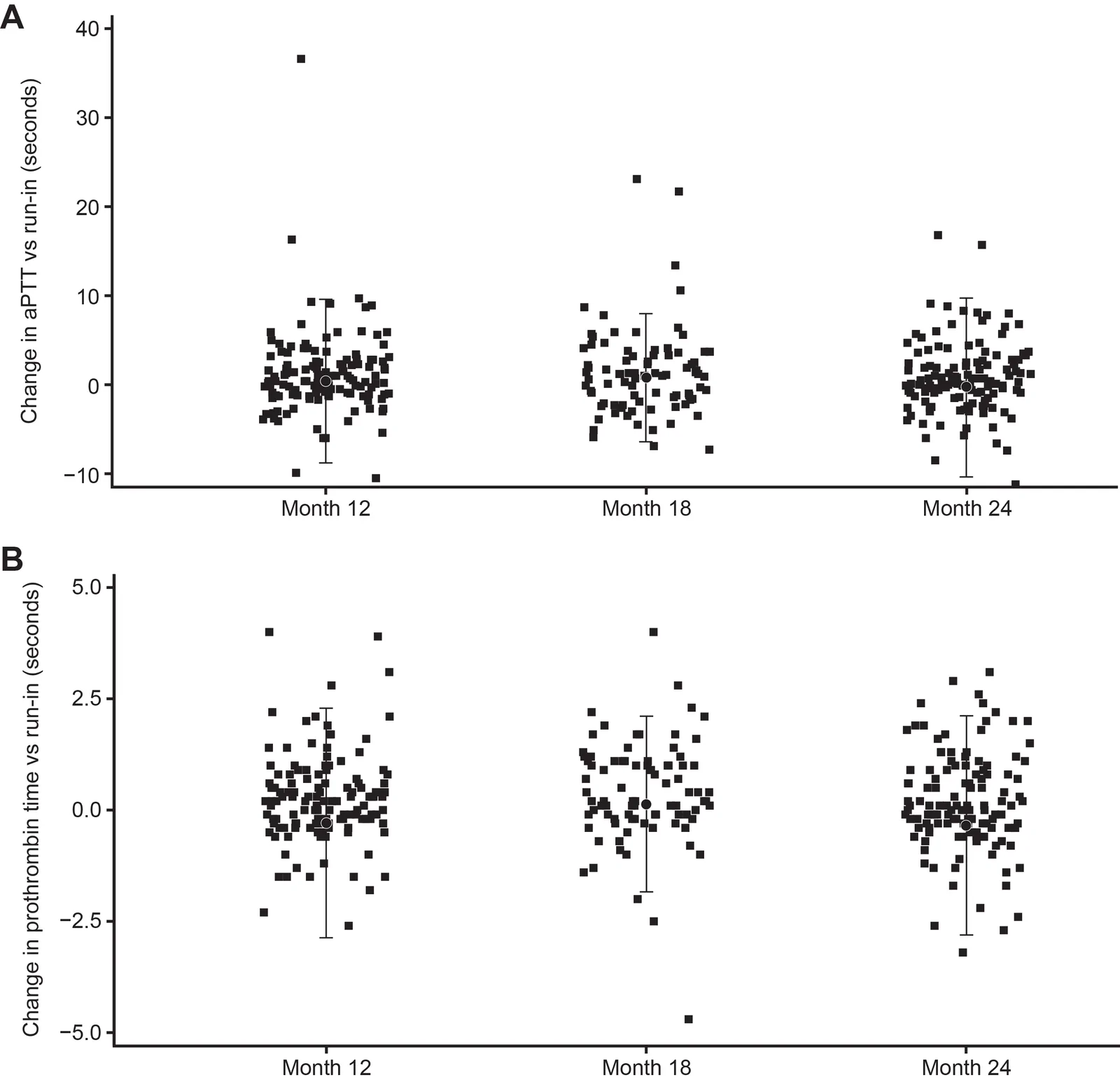
Changes in hemostasis-associated markers. (A) Change in aPTT. (B) Change in prothrombin time vs run-in in the phase 3 OLE. aPTT, activated partial thromboplastin time; OLE, open-label extension.

No garadacimab-related abnormal bleeding events were identified in patients with HAE or severe COVID-19, and laboratory markers of coagulation remained unchanged vs run-in in the HAE phase 3 OLE (Figure 1A, B). Considering the absence of reported AEs, the absence of events identified by SMQ analysis, the lack of association between laboratory markers and coagulation events, the absence of subclinical changes in laboratory markers of coagulation, and the lack of coagulation event trends in relation to garadacimab treatment, these findings indicate no impact of garadacimab-mediated FXIIa inhibition on hemostasis or bleeding risk.

The wider role of FXII and FXIIa in coagulation has been explored in patients with congenital FXII deficiency, including those with homozygous deficiency and 0 U/mL FXII clotting activity and those with heterozygous deficiency and detectable clotting activity of <0.01 to 0.8 U/mL, as well as in patients with thromboembolic disease [5], [8]. Consistent with this lack of increased bleeding risk, garadacimab-mediated FXIIa inhibition did not impact hemostasis [2], [5], [6], [7], [8].

These observations are supported by FXII knockout animal models [6], [24], [25] and models of FXII deficiency [25], [26], [27], depletion [28], and inhibition [29], in which no bleeding phenotype was reported. Preclinical garadacimab studies showed that inhibition of FXIIa with the parenteral 3F7 antibody neither increased wound bleeding nor impaired hemostatic capacity in animal models [22], supporting the observed lack of bleeding events in the clinical program.

Taken together, the presented evidence highlights the lack of impact of FXIIa inhibition on hemostasis and contributes to the understanding of the role of FXIIa *in vivo*. As postulated by Renné et al. [2], [6], FXII may contribute to the pathologic phase of clot formation (eg, with concomitant thromboembolic diseases or vessel injury) without affecting normal blood coagulation *in vivo*. This may be due to redundancy in the molecular pathways accounting for any loss in FXII activity, based on evidence from animal models [2], [6], [24], [25], [30]. Furthermore, interference with polyphosphate, which is released by procoagulant-activated platelets and induces FXII-mediated contact activation, only affects thrombosis, suggesting that FXIIa may play a separate role in hemostasis despite its important role in thrombosis *in vivo* [2].

A strength of this analysis was that hemostasis-related outcomes were assessed in a multidisciplinary manner across 2 indications in patients receiving garadacimab with increased bleeding risk due to COVID-19-associated prothrombotic risk with concomitant anticoagulant/antiplatelet medication or invasive surgical/medical procedures. A limitation was that this analysis was not designed to test this specific hypothesis; therefore, both prespecified and post hoc analyses were included.

## 4 Conclusion

A deeper investigation of hemostasis using multiple methodologies across multiple studies revealed no impact of long-term inhibition of FXIIa with garadacimab in patients at increased risk of bleeding. No garadacimab-related abnormal bleeding events were observed in clinical studies or subgroup analyses in patients with HAE or COVID-19, and concurrent SMQ analysis confirmed that identifiable bleeding events were unrelated to garadacimab. Additionally, trends in relevant laboratory markers reflected natural variation and indicated no abnormality. Together, these results confirm that long-term inhibition of FXIIa by garadacimab does not impact hemostasis regardless of baseline bleeding risk, providing valuable information for clinicians and patients and aligning with the observed lack of bleeding phenotype in the presence of congenital FXII deficiency.
